# Therapeutic nanoplatforms and delivery strategies for neurological disorders

**DOI:** 10.1186/s40580-018-0168-8

**Published:** 2018-11-30

**Authors:** You Jung Kang, Eric Gerard Cutler, Hansang Cho

**Affiliations:** Department of Mechanical Engineering and Engineering Science, Center for Biomedical Engineering and Science, Department of Biological Sciences, The Nanoscale Science Program, University of North Carolina at Charlotte, Charlotte, NC USA

**Keywords:** Nanomedicine, Nanoparticle, Drug delivery, Targeted delivery, Neurological diseases, Central nervous system (CNS), Blood–brain barrier (BBB), Biomarker, Glioblastoma (GBM), Alzheimer’s diseases (AD), Parkinson’s diseases (PD)

## Abstract

The major neurological disorders found in a central nervous system (CNS), such as brain tumors, Alzheimer’s diseases, Parkinson’s diseases, and Huntington’s disease, have led to devastating outcomes on the human public health. Of these disorders, early diagnostics remains poor, and no treatment has been successfully discovered; therefore, they become the most life-threatening medical burdens worldwide compared to other major diseases. The major obstacles for the drug discovery are the presence of a restrictive blood–brain barrier (BBB), limiting drug entry into brains and undesired neuroimmune activities caused by untargeted drugs, leading to irreversible neuronal damages. Recent advances in nanotechnology have contributed to the development of novel nanoplatforms and effective delivering strategies to improve the CNS disorder treatment while less disturbing brain systems. The nanoscale drug carriers, including liposomes, dendrimers, viral capsids, polymeric nanoparticles, silicon nanoparticles, and magnetic/metallic nanoparticles, enable the effective drug delivery penetrating across the BBB, the aforementioned challenges in the CNS. Moreover, drugs encapsulated by the nanocarriers can reach further deeper into targeting regions while preventing the degradation. In this review, we classify novel disease hallmarks incorporated with emerging nanoplatforms, describe promising approaches for improving drug delivery to the disordered CNS, and discuss their implications for clinical practice.

## Introduction

It has been challenge to improve prognose of diseases, such as brain tumor, Alzheimer’s diseases (AD), and Parkinson’s diseases (PD) [1–3] in a central nervous system (CNS) compared to other body parts. An enormous effort has been made for discovery and development of CNS drugs that can elude toward new and effective treatments; however, more than 90% of newly proposed drugs have failed to receive clinical approvals from the US Food and Drug Administration (FDA) [4]. One of major difficulties for the development of CNS treatments is the presence of a tighten blood–brain barrier (BBB) limiting drug entry to the CNS region [5]. Additionally, non-targeted delivery of diagnostic reagents or therapeutic drugs can cause detrimental effects on the neuronal cells and glial cells, extremely sensitive cellular components in the CNS maintaining brain functions and homeostasis. In this regard, it is an urgent issue to design and develop novel delivery platforms bearing the diagnostic reagents or therapeutic drugs for the neurological disorders.

Nowadays, nanomedicines in various medical fields hold a great promise to deliver the imaging agents or treatments for CNS diseases due to their beneficial features. The size of nanomedicines, operating in the range of 10–200 nm, achieve not only increased physiological properties of reactivity, strength, surface area, sensitivity, and stability [6], but also enhanced penetration properties across a BBB and deep into diseased brain tissues [7]. The drug encapsulation can prevent the degradation of drugs in the process of delivery, and the controlled release offers a safely delivery system by achieving the delivery of cytotoxic drugs to the disease area with appropriate dosage without harming healthy tissue. The modification on the surface of nanomedicines further enhance the BBB penetration as well as disease-targeting efficiency. Recently developed versatile nanomedicines can serve both diagnostic and multimodal treatment functions at the same time and proven their excellent potentials for targeting CNS diseases; a number of optimizations are still required for the future clinical purpose.

In this review, we discuss the emergence of nanomedicines for treating CNS diseases and their recent progress as well as future potentials. This paper includes the brief description of neurological diseases with signature markers widely employed in the nanomedicine area, the list of currently developed nanomedicines applied for drug delivery and sensing purposes, and promising strategies to improve BBB penetration and/or disease-targeting efficiency of nanomedicines. At the end, we briefly discuss the potentials of nanomedicines and future directions to achieve success in the clinical trials.

## List of CNS diseases and diagnosis/treating strategies

### Glioblastoma (GBM)

GBM is the most common and deadliest form of the primary brain tumor since the median survival rate is 14 months, even following aggressive multimodal therapy, such as surgery, radiation, chemotherapy, and their combined treatments [1]. This indicates the urgent need for the development of an effective strategy to eliminate brain tumors. Through extensive studies on the discovery of novel markers highly expressed on the brain tumors, their application in several nanomedicines functionalized with targeting ligands significantly succeed on improving the elimination of brain tumors [8–17]. Recently, immunotherapy targeting immune checkpoint receptors expressed on adaptive immune cells enhancing immune surveillance has emerged as another anticancer approach and led to explosion of clinical trials [18]. The biomarkers targeting the brain tumors or immune cells are summarized in Table 1. However, the intratumor heterogeneity varying between individual patients reduces the targeting efficiency and fails to eliminate the tumors completely causing the recurrence. Thus, the development of promising nanomedicines equipped with the innovative targeting and the multimodal treatment systems is now highly required for removal of cancers completely.

**Table 1:** List of CNS diseases and current strategies targeting the disease hallmarks

	Biomarker	Targeting strategy	Purpose	Examples	Refs.
GBM	MGMT methylation	Methylation-primer	Diagnosis	PCR-mMGMT	[149]
	PDGFRA mutation	PDGFRS-primer	Diagnosis	qPCR, qPCR-SSCP	[150]
		PDGR inhibitor	GBM treatment	Sorafenib Matinib Tandutinib Dasatinib	[151]
	IDH mutation	Anti-R132H-IDH1	Diagnosis	Immunohistochemistry	[152]
	EGFR mutation	Anti-EGFRvIII	Diagnosis	Immunohistochemistry	[153]
		EGFR inhibitor	GBM treatment	Erlotinib Nimotuzumab Gefitinib Cetuximab	[151]
	PD-1	PD-1 inhibitor	GBM treatment	Nivolumab Pembrolizumab	[18]
	CTLA-4	CTLA-4 inhibitor	GBM treatment	Ipilimumab	[18]
AD	Aβ oligomer	Aβ aggregation inhibitor	AD treatment	Tramiprosate	[21]
	Aβ plaque	Aβ aggregation inhibitor	AD treatment	Colostrinin	[22]
		Aβ PET ligand	PET imaging	CRANAD NIAD	[154]
	Tau	Tau aggregation inhibitor	AD treatment	Astemizole Lansoprazole	[23]
PD	DAT	Dopamine	PD treatment	L-DOPA	[27]
		Dopamine agonist	PD treatment	Bromocriptine	[28]
		D2/D3 radioligands	SPEC imaging PET imaging PET imaging	^123^I-iodolisuride ^11^C-Raclopride ^18^F-Fallypride	[155]
		Cocaine derivatives	PET imaging PET imaging	^125^I-RTI-55 ^76^Br-PE2Br	[155]
	α-Synuclein	Anti-α-synuclein	Diagnosis	ELISA	[143]

### Alzheimer’s disease (AD)

AD is a progressive and neurodegenerative brain disorder, which is the most common cause of dementia resulting in loss of memory, thinking and language skills, and behavioral changes [19]. However, no definitive cure for AD exists due to lack of knowledge of its molecular and intercellular mechanisms. Key signatures of AD progression include deposition of amyloid-beta (Aβ) peptides, neurofibrillary tangle formation of phosphoric tau proteins, and detrimental neuroinflammation in brains leading to synaptic impairment and neuronal loss [20]. The Aβ plaques around AD brains, soluble Aβ in the cerebrospinal fluids (CSFs), tau proteins presenting in the CSF are major biochemical markers, which have been targeted for the diagnosis purposes. Current therapeutic approaches are focusing on the inhibition of Aβ plaque/tau tangle formation and neutralization of their aggregations around neurons (Table 1) [21–23]. Other clinically approved drugs can only alleviate symptoms and delay AD progression by providing neurotransmitters, which promote interactions between neurons in AD brains [24]. In this regard, the discovery of novel AD markers and development of nanomedicines targeting the AD markers have been on-demand.

### Parkinson’s disease (PD)

PD is the second common neurodegenerative disease occurring primarily in the substantia nigra causing development of bradykinesia and tremors of cardinal motor functions [3]. The major hallmark found in PD models is the decreased level of dopamine transporters (DATs), which play curial roles in the uptake of dopamine by dopaminergic neurons and proceed the communications between neurons; therefore, the reduced dopamine delivery is contributed to the significant loss of neuronal functions [25]. Another marker is the accumulation of α-synuclein in the Lewy bodies, but their underlying mechanisms leading to PD dementia are not clearly defined yet [26]. These two markers have employed for PD diagnosis and treatment and are summarized in Table 1. Unfortunately, there is no current cure for the PD condition, but the treatment of initial PD stages using levodopa (L-DOPA), the precursor of dopamine, or L-DOPA agonist is the typical way to retard the progression of PD [27, 28]. However, untargeted delivery of L-DOPA can attack the peripheral system causing the adverse cardiovascular effect and dyskinesia [29]. Therefore, the neurotransmitters for the PD treatment should be encapsulated in the proper delivery system that allows the penetration of BBB, not other peripheral vessels.

## Classification of nanomedicine platforms

### Lipid-based nanoparticles: liposomes and exosomes

**Fig. 1:**
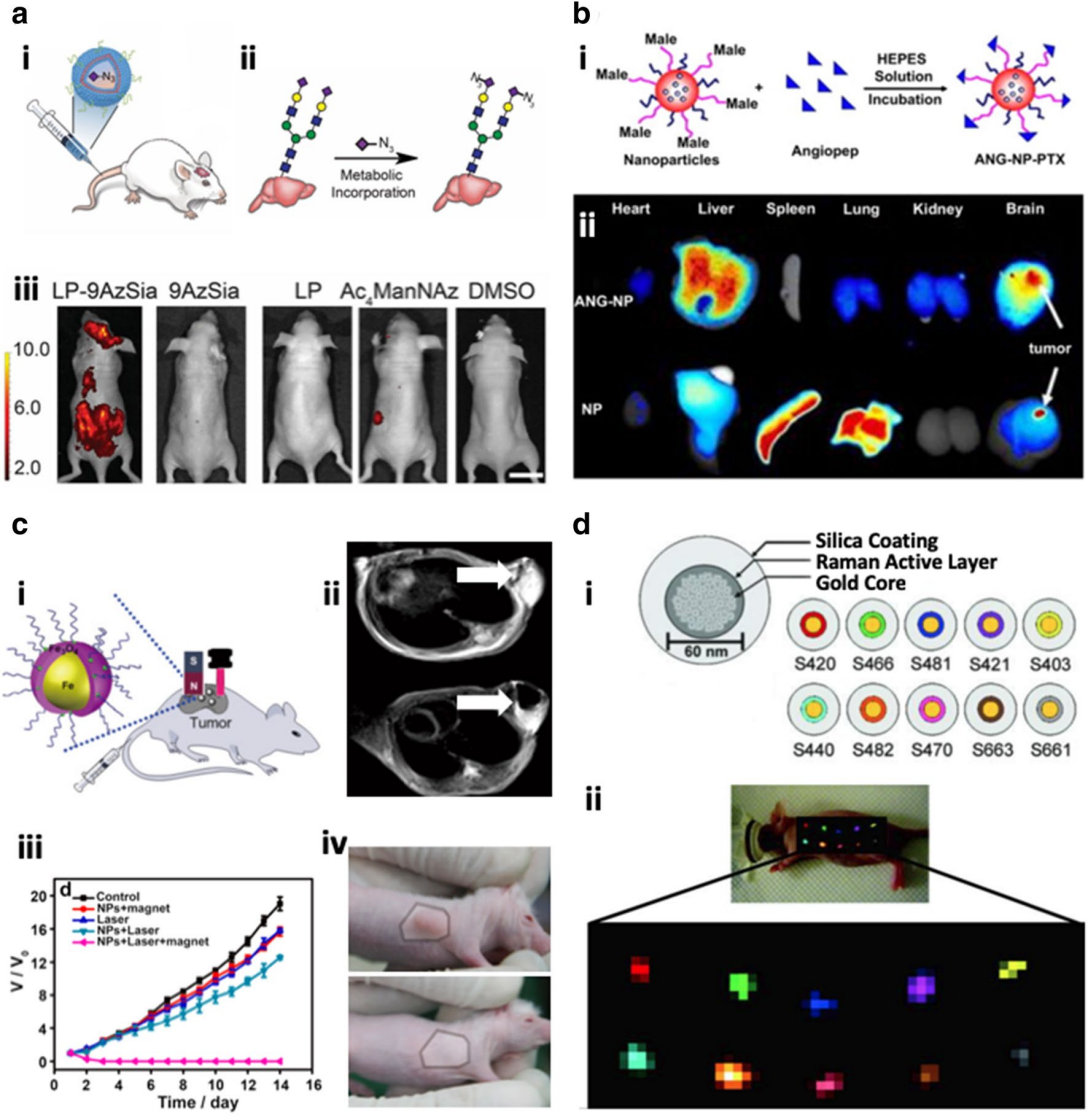
Class of nanomedicines applied in medical fields. **a** Nanoliposome used for the delivery of therapeutic drugs or imaging agents. i Schematic illustration of PEGylated liposomes bearing 9AzSia that can be injected i.v. through the tail vein. ii Schematic description of 9AzSia incorporation mechanism into the brain tissue through the binding to sialic acid. iii The liposomes bearing 9AzSia were significantly accumulated in the brain tissue compared to other conditions. Reproduced with permission [36]. Copyright 2016, PNAS. **b** Polymeric nanoparticles employed for the targeted drug delivery. i Schematic representation of paclitaxel-loaded Angiopep-PEG-PCL nanoparticles. ii Angiopep-conjugation increased targeting efficiency of brain tumors. Reproduced with permission [62]. Copyright 2011, ELSEVIER. **c** Multimodal iron oxide nanoparticles equipping both MRI and near-infrared PTT techniques. i Cartoon illustration of PEGylated Fe_3_O_4_ nanoparticles along with magnets under the magnetic field or laser irradiation for MRI or PTT. ii MRI images of HeLa bearing tumor mice without (upper) or with magnets (bottom). iii, iv Complete elimination of tumors in mice receiving magnetic targeted PTT. Reproduced with permission [90]. Copyright 2014, ELSEVIER. **d** Gold nanoparticles achieving multiple colored-imaging. i Schematic of SERS nanoparticles, a gold core coated with a Raman active layer. Depending on the type of Raman layer, the nanoparticles emit different colors. ii Combined images of multiplexing 10 different SERS nanoparticles confirmed in in vivo mice models. Reproduced with permission [104]. Copyright 2009, PNAS

Nanoliposomes, the nanosized and spherical forms of enclosed lipid bilayers, have extensively applied as a nanocarrier of therapeutic drugs, imaging agents, and genes for treatment and/or diagnosis of CNS diseases [8–11, 30, 31]. The liposomes are composing of a hydrophilic core and one or more hydrophobic spaces surrounded by lipid bilayers, and this unique amphiphilic structure enables encapsulation of both hydrophobic and hydrophilic compounds. Hydrophilic therapeutic drugs and DNAs have been loaded in the core space of nanoliposomes, which have been applied for the delivery of these compounds to CNS diseases area [11]. Moreover, various hydrophobic compounds have been entrapped into the hydrophobic interface between the lipid bilayers and applied in therapeutic as well as diagnostic purposes [10]. The common method for the nanoliposome preparation is the lipid film rehydration method, followed by either freezing–thawing cycles or sonication [32]. To yield an unilamellar liposomal suspension with a low polydispersity, this method has been combined with the extrusion technique equipping a porous membrane (pore size < 0.2 µm), which defines the size of nanoliposomes [33]. To further precisely control the size of nanoliposomes, several sophisticated techniques, such as microfluidic-based platforms, has been combined with the traditional nanoliposome preparation methods [34]. The major advantages of nanoliposomes are their high biocompatibility, excellent flexibility to tune the biophysical and physiochemical properties, and well-established preparation methods [35]. In order to achieve improved blood circulation and targeting efficiency, the surface of nanoliposomes has been further decorated with polymers (Polyethylene glycol (PEG), etc.) (Fig. 1a) [36], polysaccharides (dextran [37], mannose [38, 39], etc.), targeting molecules (transferrin [10], T7 peptide [11], Interleukin- [9, 13] Arginyl-glycyl-aspartic acid (RGD)-based peptides [10], etc.), or aptamers (GBM128 [40], etc.). The list of nanoliposomes for targeting CNS diseases are summarized in Table 2. Despite the described improvements, circulation life-time of nanoliposomes in the blood is still short, and the leakage of compounds through the lipid bilayers and the low stability toward mechanical shear stress as well as osmotic pressure have remained as major obstacles for the clinical uses [41].

**Table 2:** List of nanomedicines targeting CNS diseases

	Targeting disease	Loading molecule	Purpose	Material	Targeting strategy	Refs.
LBNP	GBM	Doxorubicin, vincristine	Anticancer	Liposome	T7 peptide	[11]
		Doxorubicin	Anticancer	Liposome	Chlorotoxin	[8]
		Doxorubicin	Anticancer	Liposome	IL-13	[9]
		Coumarin-6	In vivo imaging	Liposome	RGD, Transferrin	[10]
	AD	si-BACE	AD treatment	Exosome	Lamp-2b	[50]
	PD	Dopamine HCl	PD treatment	Liposome	–	[30]
		L-DOPA	PD treatment	Liposome	Chlorotoxin	[31]
PNP	GBM	Docetaxel	Anticancer	PEG‐PCL	AS14111 aptamer	[12]
		Paclitaxel	Anticancer	PEG‐PLGA	AS14111 aptamer	[13]
		Paclitaxel	Anticancer	PLGA	Transferrin	[14]
		Paclitaxel	Anticancer	PEG-PLGA	Pep-1	[15]
		Paclitaxel	Anticancer	PEG-PCL	Angiopep	[62]
	AD	Curcumin	AD treatment	PBCA	ApoE3	[63]
		–	PET	PBCA	^125^I-clioquinol	[64]
	PD	α-synuclein	PD treatment	PBCA	ApoE	[65]
		L-DOPA	PD treatment	PLGA	–	[59]
		Nicotine	PD treatment	PLGA	–	[60]
		Ropinirole-HCl	PD treatment	Chitosan	–	[58]
IO-NP	GBM	–	MRI	PEG-ION	Chlorotoxin	[84]
		–	MRI	Dex-SIPON	Chitosan	[86]
		–	MRI/TEM	PEG-ION	Anti-EGFRviii	[85]
	AD	–	MRI	SPION	Anti-AβPP	[88]
		–	MRI	Dextran-ION	Anti-ferritin	[89]
	PD	Rhodamine-B	MRI	MION	–	[156]
Au-NP	GBM	Phthalocyanine 4	PDT	PEG-Au-NP	Transferrin peptide	[16]
		–	PTT	PEG-Au-NP	–	[105]
		–	PTT	PEG-Au-NP	RVG29 peptide	[17]
	AD	Polyoxometalate	AD treatment	Au-NP	–	[138]
	PD	Si-α-synuclein	PD treatment	Au-NP	Chitosan	[157]

Exosomes are recently proposed nanoliposomes derived from the cellular endosomes, and they can encapsulate drugs and target CNS diseases upon the harvesting exosomes followed by post modifications that are similar to the liposome techniques [42]. The exosomes recently received great attentions as they can closely mimic membrane compositions of host cells by presenting the cell-driven surface moieties; thus, they naturally possess potentials to target diseased cells that overcome biological barriers including BBB and targeted-cellular membranes [43]. One of the outstanding cell membrane-mimicking features of exosomes, which can be applied for CNS drug delivery, is its ability to inherently express antiphagocytic markers (e.g., CD47) that endows the capability to evade monocytes and macrophages of the reticuloendothelial system (RES) reducing immune response and their clearance rate in the blood [44]. Recent studies showed that administration of drug-loaded exosomes to the mouse body or brain had significantly decreased neuroinflammatory activity and reduced septic shock compared to liposaccharide (LPS)-injected mice [45, 46]. Moreover, the enhanced endocytosis, driven by the interactions between targeting receptors on the cells and naturally expressed ligands on the exosomes, could overcome multi-drug resistance by the drug efflux transport pump such as P-glycoprotein (P-gp), which rejects drugs outside of cells and educes drug efficacy [47]. These exosomes have been decorated with active-targeting ligands (iRGD [48, 49], etc.), anchoring molecules (Lamp-2b [50], etc.), and stealth polymers (PEG [51], dextran sulfate [52], etc.) to further boost targeting efficiency, cellular uptake, and circulation half-life. However, the low mechanical stability is still an issue to be solved in order to employ them to nanomedicine platforms for CNS diseases.

### Polymeric nanoparticles (PNPs)

Solid polymeric nanoparticles typically represent nanosized and homogeneous spherical structures composed of biocompatible and biodegradable polymers, which encapsulate drugs inside of the particles or attached them to the surface of particles [53]. The polymeric nanocapsule is another form of polymeric nanoparticles having an internal cavity surrounded by polymeric shell that entraps drugs [54]. Encapsulation of drugs is highly affected by interactions between polymer and drug molecules. When the polymer–drug interaction is dominant than drug–drug interaction, drugs can be stably incorporated in the polymeric nanoparticles while drug crystallization will happen when the polymer–drug interaction is weaker than drug–drug interaction. To improve the drug encapsulation efficiency in the polymeric nanoparticles, various natural polymers (albumin [55], alginate [56], gelatin [57], chitosan [58], etc.) and synthetic polymers (poly(lactic-*co*-glycolic acid) (PLGA) [13–15, 59, 60], poly(lactic acid) (PLA) [61], poly(caprolactone) (PCL) [12, 62], poly(butyl cyanoacrylate) (PBCA) [63–65], poly(d,l-lactic acid) (PDLLA) [66], etc.) have been employed and widely applied in medicine and sensor areas. The amphiphilic block copolymers can be employed to form a nanosphere consisting of a hydrophobic solid core with a hydrophilic outer shell. This unique structure not only offers a versatile encapsulation efficiency for both hydrophobic drugs in the core and hydrophilic molecules in the outer shell but also improves the water solubility of nanospheres. Polymeric nanoparticles can be synthesized by several methods depending on the physicochemical characteristics of the compounds [53]. One of the most popular method is either oil-in-water (o/w) or water-in-oil (w/o) emulsion technique to produce nano-sized oil droplets encapsulating hydrophobic drugs in the water or vice versa [56]. Nanocapsules can be prepared by water-in-oil-in-water (w_1_/o/w_2_) double emulsion technique [66]. The nanoprecipitation, also known as a solvent displacement method, was introduced to control over the size of nanoparticles by maintaining conditions for the dropwise addition of the mixture of polymer and drugs to an aqueous solution forming nano-sized droplets in the water phase [67]. The prominent advantage of solid nanoparticles is a mechanical stability contributing to a long blood circulation time than liposomes [53]. The additional modification with PEG provides stealth property that further increases the half-life of nanoparticles [12, 13, 15, 61]. Another beneficial property is the flexible outer layer that offers multiple conjugating sites for an efficient targeting. For instance, Xin et al. showed that the tailored PCL nanoparticles exhibiting Agiopep, which target lipoprotein receptors (LPRs) on the BBB and glioma cells increasing the drug delivery efficacy, especially for GBMs (Fig. 1b) [62]. Other examples are summarized in Table 2. However, a critical issue for PNPs is a rapid initial release of cargo from the nanoparticles, called a burst effect that attributes to weak interactions of drug to polymer.

### Magnetic iron oxide nanoparticles (IO-NPs)

Magnetic nanoparticles have exclusively applied in separation and sensing of biological molecules, targeted gene/drug delivery, clinical diagnosis via magnetic resonance image (MRI), and therapy via magnetic fluid hyperthermia (MFH) [68]. The most widely applied magnetic nanoparticles are iron oxides-based nanoparticles, such as Fe_3_O_4_ (ferrimagnetic, superparamagnetic when the size is less than 15 nm), α-Fe_2_O_3_ (hematite, weakly ferromagnetic or antiferromagnetic), γ-Fe_2_O_3_ (maghemite, ferrimagnetic), FeO (wüstite, antiferromagnetic), ε-Fe_2_O_3_, and β-Fe_2_O_3_. Fe_3_O_4_-based nanoparticles. They have emerged as favorable candidates, particularly for biomedical applications due to their remarkable biocompatibility and hyperparametric characteristics [69]. In order to synthesis the iron oxide nanoparticles with controlled size and shape, high stability, and improved biocompatibility, a number of preparation methods have been proposed in the last decades. The synthesis techniques involve co-precipitation [70, 71], thermal decomposition [72, 73], hydrothermal synthesis [74, 75], microemulsion [76, 77], sonochemical synthesis [78, 79], electrochemical synthesis [80], laser pyrolysis [81], and microorganism-mediated synthesis [82]. Iron oxide nanoparticles have desired mechanical properties besides novel electrical, magnetic, and optical properties. The superparamagnetic nanoparticles are tailored to obtain excellent imaging properties to be used as MRI contrast agents since the MR signal intensity is significantly modulated without any decrease in the T1 and T2 relaxation times by surrounding water protons [83, 84]. Moreover, magnetic iron oxide nanoparticles have been PEGylated to improve bioavailability and biocompatibility by reducing oxidation and aggregation [84, 85]. The carboxylation-polysaccharide coating (dextran [86], ferumoxytol [87], etc.) significantly reduced clearance rate in blood and increased half-life of iron oxide nanoparticles. The surface modification with targeting molecule (chlorotoxin [84], chitosan [86], anti-AβPP [88], anti-ferritin [89], anti-EGFRvIII [85], etc.) allows the increased deposition in the CNS diseases. Moreover, the coating or recruiting the inorganic metallic layer offers additional physicochemical properties, such as magneto-optical properties, magnetic-electrical properties, and magnetic-thermal properties. For instance, the administrated PEGylated Fe_3_O_4_ nanoparticles along with Nd–Fe–B magnet equipping multimodal functions as MRI and near-infrared photothermal therapy (PTT) to successfully achieve the elimination of tumors (Fig. 1c) [90]. Gold coating system provides not only the stability to the NPs preventing oxidation in water but also additional binding sites (thiol group) for biological molecules at the NPs surface [91]. Other advanced metallic nanoparticles are briefly organized in Table 2. Despite such incredible features allowing both sensing and treating CNS diseases, the toxicity found in certain types of neuronal cells is still the major concern [92].

### Metallic gold nanoparticles (Au-NPs)

Metallic gold nanoparticles have been extensively applied in the wide range of biomedical science and engineering areas. These nanoparticles have been intensively applied in diagnostic imaging systems, where most states have been described as the metallic nanoparticle-mediated imaging modalities by using MRI, computed tomography (CT) scan, Positron-emission tomography (PET), ultrasound, and surface-enhanced Raman spectroscopy (SERS) imaging systems nowadays [93]. The most popular method to synthesis gold nanoparticles is the citrate reduction of gold tetra-chloric acid, which is low-cost and performed under mild preparation environments [94]. Other preparation methods include chemical reduction [95, 96], photochemical synthesis using UV irradiation [97, 98], sonochemical synthesis [99], laser ablation [100], seed mediated growth [101], and sonoelectrochemical synthesis [102]. Gold nanoparticles especially have received great attentions as promising biomaterials in bio-nanotechnology and related areas due to their unique optical and thermal properties as well as ease of surface functionalization, which enable targeted delivery of genes/proteins/drugs and various biological assays. The optical property mostly attributes to the localized surface plasmon resonance (LSPR), which absorb and emit various light colors depending on size, shape, local reflective index, and aggregate status of nanoparticles (Fig. 1d) [103, 104]. As increasing in the aspect ratio forming gold nanorods, they can absorb from visible up to Infra-red region and obtain increased oscillator strength, which allows PTT. Numerous studies have developed gold nanoparticles functionalized with targeting molecules for brain cancers and BBB and applied them for the diagnosis and treating purposes. Especially, the targeted gold nanoparticles are beneficial for the PTT ablation of brain tumors; upon reaching to the brain tumor parts, the laser light at the IR region can pass through the skin, warm-up the tumor part, and specifically kill the cancer while sparing healthy tissues [16, 17, 105]. Recently, gold nanoshells and nanocages are developed for further application to high-resolution imaging techniques such as MRI [69]. Currently developed gold nanoparticles for the CNS diseases are summarized in Table 2. However, the long-term cytotoxicity is a major limitation to be solved in the future [106].

## Promising strategies to improve drug penetration into BBB

**Fig. 2:**
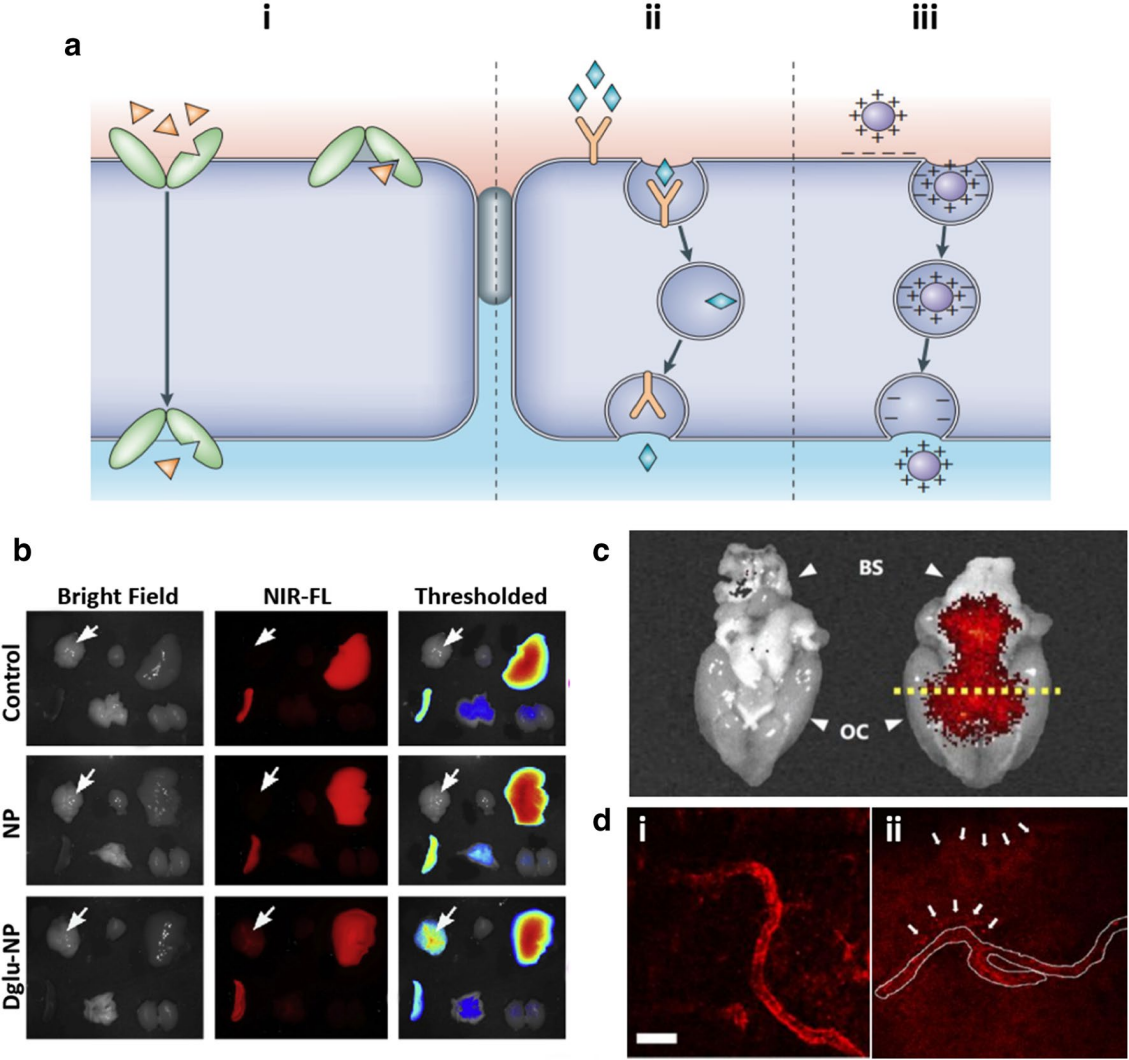
Strategies for improving nanomedicine delivery passing through BBB. **a** Schematic representation of three major mechanisms to enhance BBB penetration: i transporter-mediated transcytosis, ii receptor-mediated transcytosis (RMT), and iii absorption. Reproduced with permission [5]. Copyright 2006, Nature Publishing Group. **b** Nanomedicines functionalized with transporter-targeting molecules. Glucose transporter-targeting 2-deoxy-d-glucose decoration increased the deposition of nanoparticles in the brain passing through BBB while fluorescent dye, DiR, encapsulation allowed an efficient imaging. Reproduced with permission [115]. Copyright 2006, ELSEVIER. **c** Absorption-enhancing nanoparticles. The magnetic nanoparticles decorated with albumin can be delivered to the brain region from the vein through the enhanced absorption pathway. Reproduced with permission [109]. Copyright 2016, Nature Publishing Group. **d** Ligand-targeted nanoliposomes promoting receptor-mediated endocytosis. In vivo multiphoton images of brains receiving i non-targeted liposomes or ii transferrin-functionalized liposomes. The targeted liposomes were able to pass the BBB and accumulated in the brain regions via RMT pathway while non-targeted liposomes were concentrated in the vessel regions. Scale bar represents 25 μm. Reproduced with permission [122]. Copyright 2018, Nature Publishing Group

The blood–brain barrier is a tighten mechanical barrier between blood streams and brain tissues, which is highly selective and only permits the entrance of essential water, nutrients, and neurotransmitters governing the maintenance of CNS homeostasis and modulation of neuronal signal propagation [5]. Moreover, this barrier regulates the immune cell transports to maintain the intracranial pressure but limit the entry of toxins and pathogens circulating in the blood to prevent potential neuronal damages. The presence of this unique and restrictive barrier is the major limitation to deliver therapeutic drugs or imaging compounds to CNS regions. This barrier has known to reject the entrance of 98% of small-molecule drugs and ∼ 100% of large-molecule drugs to the CNS [107]. To achieve a successful delivery of nanomedicines to the CNS across the BBB, a number of recent studies have investigated to understand the characteristics of BBB transports and adopt the mechanisms on the design of nanomedicines. There are five major pathways to permit the entrance of molecules across the BBB: (1) paracellular pathway through tight junctions for hydrophilic molecules, (2) transcellular diffusive pathway for hydrophobic molecules, (3) transporters-mediated specific molecules (4) receptors-mediated transcytosis of ligands or ligand-conjugated carriers, and (5) absorption of positive charged molecules [5]. Among the pathways, most nanomedicines select strategies of encapsulating drugs in the adsorptive molecules promoting passive transports or nanomedicines conjugated with transporters or receptors improving active transports across the BBB (Fig. 2a) [5].

### Passive transporting system

The electrostatic interaction-mediated adsorption is a passive diffusion for small biomolecules driven by a concentration gradient across the BBB [5]. One of novel molecules promoting the passive diffusion across the BBB is albumin. Albumin is an abundant component of human blood and has been widely applied in the medical fields as a novel biomaterial due to their excellent biocompatibility, long half-life in blood (~ 20 days), and ability to pass across the BBB [108]. Moreover, the free thiol groups on albumin can offer binding sites for metal ions allowing efficient incorporation with metal nanoparticles by coating the metal surface (Fig. 2c) [109]. The albumin is especially beneficial for brain cancer treatments because of albumin binding receptors, such as SPARC and gp60, overexpressed on gliomas [55]. Various albumin-based nanomedicines have been clinically approved for drug carrier platforms targeting various diseases (e.g., diabetes, various tumors, multiple sclerosis, etc.) and precise diagnosis or bioimaging systems (e.g., SPECT, PET, MRI, ultrasound imaging, etc.) [110]. In order to apply them for the CNS diseases, cationic albumin-coated PEG-PLA nanoparticles were designed to promote albumin-mediated passive transport across BBB showing 7.9-fold increased BBB transport efficiency compared to non-charged nanoparticles [61]. However, this passive diffusion transport system has not been solely applied in the development of nanomedicines targeting the CNS, but combined with other passive or active targeting systems due to the low targeting efficiency. The albumin-based nanoparticles have been decorated with targeting antibodies against receptors expressed on brain endothelial cells such as transferrin and insulin [111, 112]. Moreover, Lin et al. improved in vitro BBB penetration of albumin nanoparticles by modification of albumin with a cell-penetrating peptide, low molecular weight protamine (LMWP), and applied the nanoparticles to co-encapsulate anticancer drugs, paclitaxel and fenretinide, and deliver them to the CNS region [55]. This study reported that LMWP-albumin nanoparticles achieved 2.5-fold increased BBB penetration compared to bare albumin nanoparticles.

Another passive transporting system can be promoted by decorating transport protein on the surface of nanoparticles [5]. Transport proteins, expressed on the endothelial cells in both the abluminal and luminal sides, modulate the influx of essential nutrients, peptides, and ions or the efflux of wastes and toxins. For instance, glucose transporters (GLUTs), highly expressed on endothelial cells, mediate the influx of sugars to the CNS by facilitated diffusion in response to the concentration gradient due to high demands on glucose and other energy sources (e.g., mannose, glucosamine, fructose, and other glucose derivatives) in the brain [113]. In this regard, the conjugation of glucose or glucose analogues, specifically targeting type 1 transporter (GLUT1), has widely applied in the nanomedicines to enhance transports across the BBB. For instance, β2-mercaptoethoxy-glucose-conjugated gold nanoparticles have proven at least 3-times higher potentials to pass the 3D cultured BBB models with hCMEC/D3 cells through binding to GLUT1, compared to glutathione-conjugated gold nanoparticles [114]. Moreover, gold nanoparticles decorated with 2-deoxy-d-glucose, which has known to increase the BBB penetration efficiency through interactions with GLUT1 (Fig. 2b) [115], produce a high-resolution and anatomic information in a single CT scan of tumors [116, 117]. However, a number of studies reported that glycosylation frequently failed to promote the BBB penetration due to random conjugation of sugars to the nanomedicines since OH groups only positioned at C1, C3, and C4 of glucose and its derivatives are actual binding sites of GLUT1 [118]. To increase transcytosis of glycosylated nanomedicines across the BBB, Anraku et al. designed PEG-micelles conjugated with C6 OH group in order to preserve the binding ability of glucose to GLUT1 [119]. The results showed that the linkage at C6 improved interactions with GLUT1 and the significantly increased BBB penetration rate compared to the linkage at C3.

### Active transporting system

The transporting system based on the receptor-mediated transcytosis (RMT) is the most efficient and promising strategy to promote BBB permeabilization [5]. The receptors expressing on the luminal side of brain endothelial cells and mediating RMT, include transferrin receptors (TRs), scavenger receptors (SRs), insulin receptors (IRs), and lipoprotein receptors (LPRs). In contrast to passive transport across the BBB, the RMT is an active transport against concentration gradient requiring large amount of energy [120]. Upon the binding of ligands on their receptors, ligands and ligand-decorated molecules/carriers undergo endocytosis via membrane invagination forming intracellular transport vesicles. The vesicles containing ligand-receptor complex are experiencing exocytosis that transports the vesicles to the basolateral side so that the vesicles are fusing with the plasma membrane and releasing the encapsulated components to the CNS. Due to the tightly regulated process of the RMT, nanomedicines conjugated with ligands initiating the RMT offers ideal platforms for selective delivery of cargo to the CNS compared to passive transporting systems. Among the ligands or antibodies targeting receptors expressed on the endothelial cells, the transferrin peptide, transferrin protein, and antibody against transferrin (anti-TRs) have been tremendously explored in terms of their ability to promote the RMT across the BBB (Fig. 2d) [121, 122]. Not only the high binding affinity to TRs but also the avidity effect, TR-targeting system has been deemed a promising strategy to enhance the RMT-mediated transport into brain parenchyma from systemic administration [123]. In this regards, a number of studies conjugated transferrin peptides or anti-TRs to various nanomedicine platforms, such as human serum albumin (HSA) nanoparticles [111], gold nanoparticles [123], and nanoliposomes [124], and proved the promoted the BBB penetration of nanomedicines. Another evident strategy is targeting IRs to promote the BBB transport. A number of studies employed antibodies against IRs (anti-IRs) instead of insulin or insulin peptides due to their notable targeting efficiency. Coloma et al. combined the anti-IRs with glial-derived neurotrophic factor peptide (GDNF), which showed great potentials to target both neurons and the BBB [125]. Ulbrich et al. decorated HSA nanoparticles with anti-IRs (29B4) and reported the improved drug delivery efficacy across the BBB [112]. To further increase the BBB penetration, nanomedicines can be tailored with dual ligand-conjugation system with two types of ligands, one for initiating transcytosis of nanomedicines across the BBB and the other for further promoting penetration mechanisms.

## Prospective approaches to enhance the targeted delivery to diseased regions

For the successful drug delivery, nanomedicines should be delivered to the specific regions around the targeted diseases [126, 127], in order to reduce unexpected cytotoxicity in the brain where most of cells governing brain functions and immune systems can be easily disrupted by external molecules. Both passive and active targeting systems can be applied for the development of CNS disease-targeting nanomedicines; however, the passive targeting system is not solely adopted excepting for the brain tumor-targeting nanomedicines. Therefore, several studies employed the combined targeting systems in order to achieve both accurate diagnosis and treatment for the specific diseased brains.

### GBM-targeting system

Passive targeting systems have been applied in the design of brain cancer-targeting nanomedicines due to the highly leaky vasculature environments around the tumors. The enhanced permeation and retention (EPR) is the unique phenomenon that allows nanomedicines loading therapeutic drugs with size range of 150–200 nm to be easily accumulated in the tumor regions passing through the vasculatures having several pores with size range of 200–500 nm [7]. The nanoparticles, equipped with the property of inducing EPR effects by controlling size below 200 nm, not only enhance the penetration to the BBB but also increase the accumulation of drugs in the solid tumors more than several-fold higher compared to free drugs. In addition, these nanoparticles are advantageous for the drug delivery using interventional procedures such as catheters. The anticancer drugs encapsulated in liposomes (Doxil1/Caelyx1, Myocet1, Daunosome1 loading doxorubicin) or polymeric nanoparticles (Abraxane encapsulating paclitaxel) are particularly designed to promote the EPR effects and recently received clinical approvals [128]. Despite the enhanced penetration toward the solid tumors, more than 95% of passively targeted nanomedicines failed to deliver their cargo to the brain tumor regions, even after multiple administration trials through the intravenous methods causing unexpected side effects such as neuroinflammation and cytotoxicity in the brain [129]. Thus, the passive targeting strategy has to be combined with another targeting strategy, such as active targeting system with tumor-specific targeting ligands [12, 130].

**Fig. 3:**
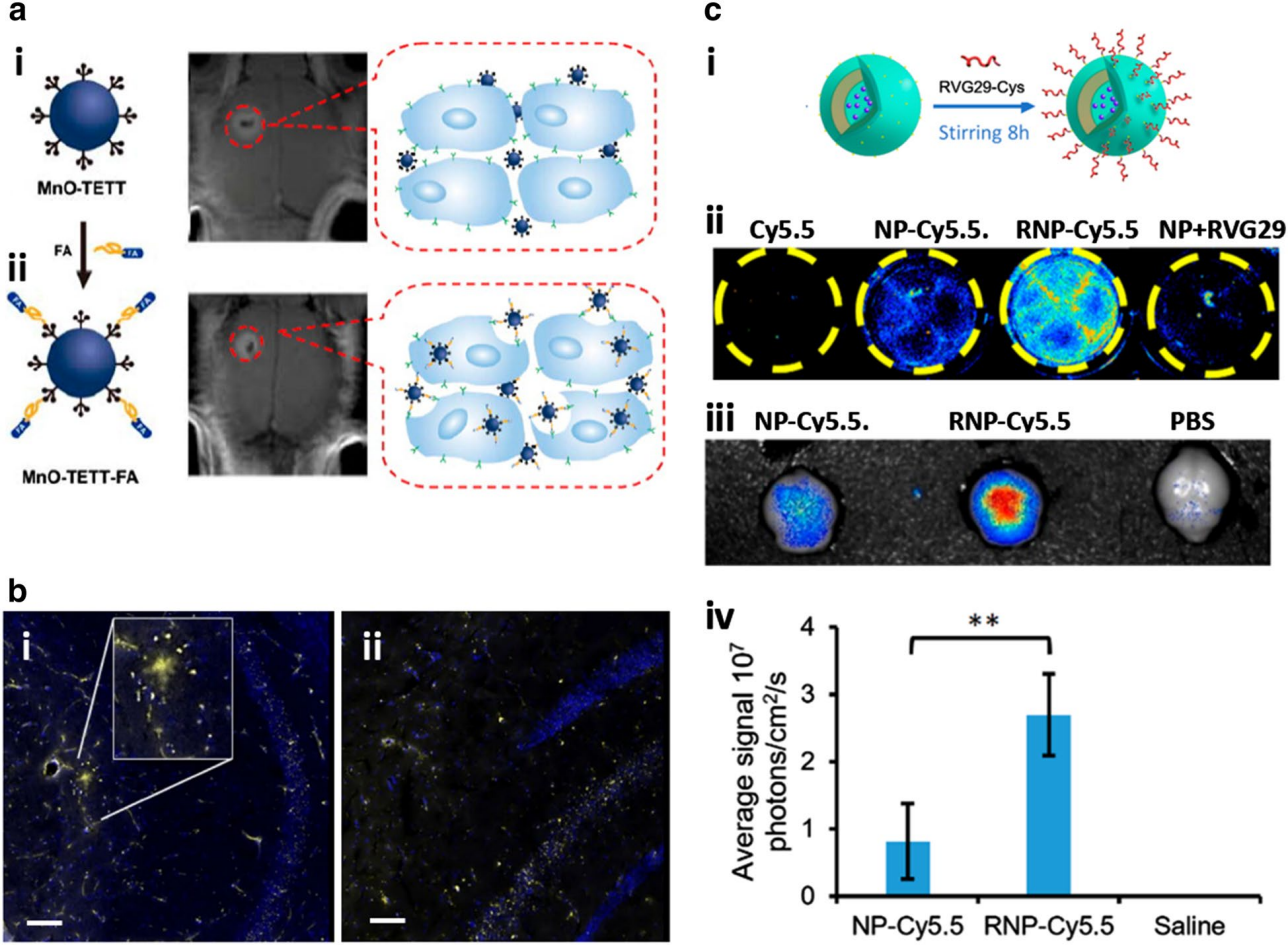
Approaches for targeting each CNS disease. **a** Nanomedicines functionalized with ligands targeting receptors expressed on GBMs. The oleic acid capped manganese oxide (MnO) oxide nanoparticles were decorated with folic acid, a glioma-specific moiety. MRI images of brains bearing C6 tumors, receiving i MnO NPs decorated with *N*-(trimethoxysilylpropyl) ethylene diamine triacetic acid (TETT) silane or ii MnO NPs decorated with both TETT and folic acid via post intravenous injection for 2 h, confirmed the deposition of targeted nanoparticles in the small tumor region. Reproduced with permission [115]. Copyright 2014, American Chemical Society Publications. **b** Aβ-targeted nanomedicines for AD diagnosis. MRI images show that i Gadolinium (Gd)-based nanoparticles decorated with Aβ-targeting peptides were accumulated around Aβ plaques in the AD mouse brain while ii bare Gd nanoparticles were not able to detect Aβ plaques. Scale bars represent 50 µm. Reproduced with permission [141]. Copyright 2016, Springer Nature. **c** Nanomedicines targeting PD. i Schematic configuration of PD-targeted nanoparticles. The conjugation of RVG29 to nanoparticles (RNP-cy5.5) achieved, ii the significantly enhanced in vitro BBB permeability and the iii, iv effective delivery to in vivo PD brain region compared to bare nanoparticles. Reproduced with permission [148]. Copyright 2018, American Chemical Society Publications

Another brain tumor-targeting strategy is the active targeting system by decorating nanomedicines with targeting ligands, which have high binding affinity to receptors or other surface membrane proteins overexpressed on targeting brain tumors [128]. The ligands include small molecules, peptides/aptamers, proteins, and antibodies as well as their fragments that specifically bind to the receptors and trigger the internalization of the decorated nanomedicines. The major advantage of the active targeting over the passive targeting is an incredible selectiveness to the specific tumors that enable the effective delivery of therapeutic drugs or diagnostic reagents to disease sites reducing local cytotoxicity [131]. As previously described, the transferrin, a gold standard targeting molecule for increasing the BBB penetration, and its moieties (e.g., TfR-lytic hybrid peptide [132], OX26 [111], anti-TR antibodies [15, 90], etc.) have been extensively established as brain targeting molecules as well and decorated on various nanomedicines. In addition, the conjugation of LDLR, LRP, or IR targeting molecules on the nanomedicines have great potentials to enhance the BBB penetration as well as internalization into brain tumors [112, 133, 134]. The hyaluronic acid (HA) and folate, two general tumor targeting molecules as they have high affinities to CD44 and folate receptors that are highly expressed on various cancer cells including brain tumors, have proven their excellent targeting efficiency of HA or folate-decorated nanomedicines to brain tumors compared to bare nanomedicines (Fig. 3a) [135, 136]. Other verified ligands to promote cell uptake by brain tumors, are IL-13 [9], RGD [130], and mutant receptors expressed on the GBMs (e.g., EGFRviii) [85]. In spite of excellent delivery efficiency, the high heterogeneity found in most brain tumors reduces the therapeutic efficacy of targeted nanomedicines [137]. To overcome such limitation, currently developed drug delivery carriers have been modified with multiple targeting molecules. The dual ligand-conjugation system with two targeting machineries can improve both penetration of nanomedicines across BBB and accumulation in brain tumors [12, 130].

### Alzheimer’s disease-targeting system

As described in the previous section, deposition of amyloid-beta (Aβ) peptides around AD neurons is the key signature of AD progression [20]. In order to deliver nanomedicines to AD brains, the nanoparticles have been decorated with ligands targeting either Aβ oligomers/plaques or associated modulators [64, 88, 89, 138]. Through the ligand-decoration, the nanomedicines can achieve the local delivery of AD treatments or imaging reagents to the disease regions surrounded by Aβ while sparing healthy brain parts. For the diagnosis of early AD via MRI, the recently developed gadolinium (Gd)-based nanoparticles having excellent imaging contrast efficiency, were conjugated with various Aβ aggregate-targeting molecule such as popular Aβ probe (e.g., Pittsburgh compound B [139], etc.) and anti-Aβ antibody (e.g., IgG4.1 [140], LPFFD [141], KLVFF [141], etc.) (Fig. 3b). Furthermore, apolipoprotein E3, a high AD-risk marker associating with Aβ depositions, has proven its targeting efficiency for AD brains, and recent study introduced ApoE3-functionalized PBCA nanoparticles to achieve the enhanced uptake by AD brains [63]. For the efficient delivery of therapeutic drugs to AD brains, Zhang et al. developed the PEG-PLA nanoparticles with TGN peptides for the enhanced BBB penetration and QSH peptides for the Aβ42-binding, which achieved 3.41-fold increased targeting efficiency for AD compared to bare nanoparticles [142]. However, Aβ-targeting system limits to detect and treat for early staged AD brains, indicating the significance of developing new targeting systems for the AD models in later stages. Therefore, nanomedicines conjugated with multiple check points involved in AD pathogenesis would address the current limitations found in AD treatments. In addition, present sensing and analytic nanoplatforms can further contribute to the discovery of novel markers targeting late-onset AD models.

### Parkinson diseases-targeting system

For the diagnosis purpose, the detection of decreased dopamine and increased acetylcholine levels in the brain tissue, bloods, urine samples are the major strategies to diagnose PD [25, 143]. To this end, the receptors or aptamers having the high affinity to the neurotransmitters are conjugated with conductive nanomedicines, which are coated on the electrode. Bardon et al. showed gold nanoparticles cross-linked with biscyclo (paraquat-p-phenylene) receptors to detect dopamine and L-DOPA achieving a detection limit of 1 × 10^−6^ M [144]. Recent studies applied the dopamine-binding aptamer-coated gold nanoparticles immobilized on the electrode to further improve the sensitivity up to 1 × 10^−8^ M, which allow to detect the decreased dopamine level from urine samples with high accuracy [145]. However, the current sensing platforms based on electrochemical property of dopamine binding to the receptors or aptamers require several steps and longer time compared to imaging techniques, such as MRI, CT, and etc.

The major strategy for the delivery of dopamine or other therapeutic molecules/DNA to promote neural viability in the PD area is improving the BBB penetration by decorating nanoparticles with transferrin or lactoferrin, which results in the accumulation of drugs in the CNS region [146]. To further target neurons, neurotransmitters binding to the dopamine D2 and D3 receptors have been proposed as useful ligands for targeting PD brains [25]. In addition, rabies virus glycoprotein (RVG) peptide targeting acetylcholine receptors and Tet1 peptide targeting GT1b gangliosides highly expressed on neuronal cells, can be used for improving the entry pathway into neurons [147]. You et al. described polymeric nanoparticles conjugated with RVG peptides and proved their great potentials to deliver the PD preventing drugs to neurons by using PD mouse models (Fig. 3c) [148]. Since there are no representative biomarkers expressed on the PD brains, the current PD-targeting nanomedicines cannot target the disease specific regions causing unexpected toxicity in the CNS. Therefore, it is an urgent issue to discover novel biomarkers highly expressed on PD neurons so that improve the targeted delivery of PD drugs or PD detecting reagents encapsulated in the nanomedicines to the disease brains.

## Conclusion and outlook

To sum up, we overviewed the currently developed nanoplatforms for prognosis purpose as well as the promising strategies for the targeted delivery of drugs to the disordered CNS regions effective for GBM, AD, and PD. Through the enormous efforts to discover biomarkers and create nanomedicines targeting the biomarkers in the past decade, a number of studies have made fruitful progress to overcome current limitations that retard the clinical translation of CNS-targeting medicines. Moreover, recently proposed hybrid nanomedicines, combined with two or more nanomedicines, have accomplished diverse functions, such as dual or multiple targeting systems, multimodal therapies, multimodal diagnosis, or their combinations. These studies have thoroughly proven the incredible abilities of hybrid nanomedicines for detecting or treating neurological disorders both in vitro and in vivo, which may lead to success on the clinical translation at the end. Therefore, the significant improvement in the nanotechnologies for synthesis and functionalization of versatile nanomedicines will contribute to the discovery of novel biomarkers and nanomedicines incorporated with the biomarkers to bring promising strategies to conquer the CNS diseases in the near future.
